# Helping people to die

**DOI:** 10.2471/BLT.24.020924

**Published:** 2024-09-01

## Abstract

As an increasing number of jurisdictions legalize assisted dying, attention is focusing on palliative care clinicians’ role in service delivery. Gary Humphreys reports.

Dr Gert Huysmans remembers the first person he assisted. “Arjen [name changed] was in his 50s, a larger-than-life character, a motorcyclist,” recalls the 62-year-old physician. “He had a cancerous tumour that had overtaken his mouth and jaw. When he reached the point that he couldn’t ride his motorcycle anymore, he got his son to put it on a trailer behind the car and rode it around like that for a while. And then he came to me.”

Huysmans provides palliative care services for people with life-limiting illnesses at the Coda and Lotus palliative care centres in Wuustwezel on the outskirts of Antwerp, Belgium.

He also provides euthanasia, a form of assisted dying.

Assisted dying takes two forms: physician-assisted suicide, in which health-care professionals prescribe lethal drugs for the patient to self-administer; and euthanasia, in which health-care professionals administer the lethal drugs themselves. With both procedures the intent is to end a patient’s life at their voluntary request, subject to eligibility criteria and requisite safeguards.

Belgium was one of the first countries to legalize assisted dying, adopting legislation in May 2002, five years after the State of Oregon did so in the United States of America (USA).

Since then, at least 15 countries, have legalized the service, some – including Canada, Colombia, Luxembourg, the Kingdom of the Netherlands, Portugal and Spain – legalizing both forms, others – including Germany, New Zealand and Switzerland – legalizing assisted suicide only. In Australia, the Northern Territory is now the only jurisdiction in the country whose residents do not have access to assisted dying, while in the USA, 11 states plus the District of Columbia have legalized assisted suicide only.

“With the ageing global population and increased prevalence of chronic diseases, interest in and demand for assisted dying has grown significantly,” explains Julie Ling, a palliative care specialist who is chief executive officer of the European Association for Palliative Care, and consults for the European Regional Office of the World Health Organization (WHO).

There are also indications of an increase in the number of people availing themselves of assisted dying services. For instance, Belgium has gone from 235 cases in the first full year after legalization to 3423 cases reported in 2023, a rising trend which Huysmans believes is partly driven by an increase in requests for euthanasia from people suffering from multiple morbidities.

“Demand for assisted dying has grown significantly.”

Julie Ling

However, as Huysmans points out, such data need to be interpreted with caution because in Belgium – as in other countries – death certificates list the underlying illness, such as cancer, as the cause of death, rather than the euthanasia procedure itself, in part to maintain patient–doctor confidentiality.

One of the striking aspects of the current regulatory landscape is the diversity in approaches taken to assisted dying. Commonalities exist, such as requirements regarding minimum age and the patient’s state of mind and capacity to take decisions, but there are also significant variations, notably regarding the mode of medication delivery.

Meanwhile, governments considering the introduction of assisted dying regulation – often against the backdrop of increasing public demand – continue to struggle with questions regarding how best to design, regulate and deliver assisted dying services. Global institutions that might be expected to generate normative guidance on these issues – including WHO – have yet to do so.

To date, one area of consensus appears to be the need to rely on palliative care clinicians to deliver such services. Huysmans understands this. “It is logical that people turn to us,” he says. “We have specialist knowledge, not only of the relevant medicines but also of caring for people who are facing the end.”

Despite this acceptance, Huysmans initially had reservations. “All of my training as a general practitioner was geared towards healing, not killing,” he explains. “And even as a palliative care specialist, my focus is end-of-life care which is not the same as ending a life.”

According to Ling, this is a view shared by many. “Most palliative care clinicians reject the idea that they should be the ones to deliver assisted dying services,” she says. “They are not trained to do it, and do not wish to be.”

Nancy Preston, a professor of palliative care at Lancaster University, who has worked extensively on assisted dying, reports similar attitudes in the United Kingdom of Great Britain and Northern Ireland.

“Many of the doctors who support legal reform in favour of assisted dying in the United Kingdom acknowledge they do not wish to be directly involved,” she says, adding that for many, the psychological impact is a core concern. “Helping someone to die is much more than just a medical procedure,” she points out. “It has a tremendous impact on the clinicians involved.”

According to Preston, clinicians’ reluctance goes beyond a personal or professional disinclination. “There is also concern about the legal and ethical issues surrounding assisted dying, including the potential exposure of patients to manipulation or undue pressure,” she says.

Sarah Barber, Director of the WHO Centre for Health Development at the WHO Kobe Centre in Japan, highlights concerns about recourse to assisted dying reflecting shortcomings in palliative and psychosocial care provision.

“Depression, anxiety and other medical conditions can often be managed through health and home-based interventions that enable people to maintain functional ability and good quality of life,” she says. “The concern is that recourse to assisted dying may reflect a lack of such interventions.” Huysmans concurs, pointing out that, “good palliative care is the best guarantee that euthanasia is not performed because of bad or absent care.”

“It is a request that I cannot simply ignore.”

Gert Huysmans

In some contexts, clinicians’ reluctance is informed by cultural prohibitions. “Opposition to assisted dying is rooted in most communities here, whether they are Christian, Muslim or observant of other African religious systems,” says Dr Emmanuel B K Luyirika, Executive Director of the African Palliative Care Association based in Uganda. “If people learned that doctors were delivering assisted dying services, the hospices would empty,” he says.

Given the complexity of the issues in play, it is perhaps not surprising that so few countries have developed coherent policy, and those that have typically take a long time to do so. For example, it took nearly two decades for the Colombian government to formalize the process through which euthanasia could be legally practised, starting in 1997 and ending in 2015, when the Ministry of Health and Social Protection of Colombia issued a resolution that provided the necessary regulatory framework.

Despite decades of established practice and lessons learned, little has changed as attested by Preston, who, in 2023, briefed a select committee convened by the United Kingdom parliament to consider the evidence regarding the impact of assisted dying on families and health-care workers. “The Chair of the Health and Social Care Committee, which launched an inquiry into assisted dying in December 2022, said that it was the most complicated thing the committee had ever looked at,” she says.

Complex though it may be, for Preston one thing is clear: responsibility should not fall on doctors alone. She advocates “de-medicalizing” the process, arguing that societal considerations and interventions should prevail. “In Switzerland, assisted dying is considered a civil rather than a medical act,” she points out. “The patient is supported, but they take the final step, usually in the company of relatives at home.”

However, she also acknowledges that volunteer doctors, predominantly affiliated with right-to-die associations such as Dignitas, perform important supportive measures such as presenting medication and delivery options.

It would seem, then, that clinicians will need to be involved, albeit on a voluntary basis, and given the necessary training.

Doctors like Gert Huysmans, who, as stated above, also initially had reservations about providing the service. His first patient helped him overcome them.

“Arjen told me he wanted to end his life,” he recalls. “It was not about managing unbearable pain. It was simply that living with his appalling disfigurement and the disability that came with it had become intolerable to him.”

This echoes the findings of Dr Linda Ganzini, a psychiatrist and researcher at Oregon Health & Science University in the USA, who reports that patients primarily seek assisted dying because of loss of autonomy and dignity, rather than due to unmanageable pain.

Huysmans recalls the day Arjen asked for his help. “He looked at me out of that terrible mask of a face and he made a throat-cutting gesture with his index finger. I will never forget it. It was a look of resolve, not desperation. And so we got organized, and I administered the injection and he died on his own terms.”

Since then, Huysmans has gone on to assist around seven people a year to end their lives. He also trains other clinicians to provide the same service. “It does not get easier,” he says. “In fact, I think as I get older, I become more sensitive to the cruelty of separation. But I deal with it because the patient asks for assistance, and it is a request that I cannot simply ignore.”

**Figure:**
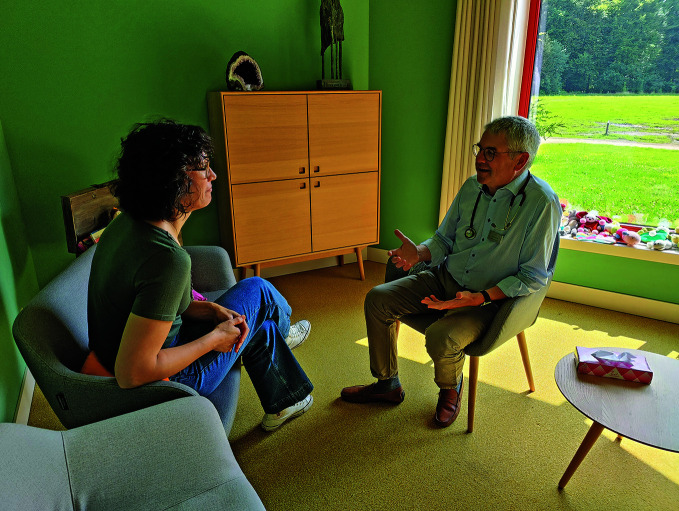
Dr Gert Huysmans in discussion with a colleague

Courtesy of Gert Huysmans

